# High glycated albumin is an independent predictor of low response to clopidogrel in ACS patients: a cross-sectional study

**DOI:** 10.1186/s12933-020-01146-w

**Published:** 2020-10-09

**Authors:** Xiliang Zhao, Quan Li, Chenchen Tu, Yong Zeng, Yicong Ye

**Affiliations:** Department of Cardiology, Beijing Anzhen Hospital, Capital Medical University, Beijing, 100029 China

**Keywords:** Glycated albumin, Acute coronary syndrome, Clopidogrel

## Abstract

**Background:**

Glycated albumin (GA) is a marker of short-term glycemic control and is strongly associated with the occurrence of diabetes. Previous studies have shown an association between GA and the effect of clopidogrel therapy on ischemic stroke. However, limited information is available regarding this relationship in acute coronary syndrome (ACS) patients. In this study, we evaluated the effect of GA on platelet P2Y12 inhibition by clopidogrel in patients with ACS.

**Methods:**

Consecutive Chinese patients with ACS who received loading or maintenance doses of clopidogrel in addition to aspirin were recruited. At least 12 h after the patient had taken the clopidogrel dose, thromboelastography (TEG) and light transmittance aggregometry (LTA) were used to calculate the quantitative platelet inhibition rate to determine clopidogrel-induced antiplatelet reactivity. A prespecified cutoff of the maximum amplitude of adenosine diphosphate (ADP)-induced platelet-fibrin clot strength > 47 mm plus an ADP-induced platelet inhibition rate < 50% assessed by TEG or ADP-induced platelet aggregation > 40% assessed by LTA to indicate low responsiveness to clopidogrel were applied for evaluation. Patients were categorized into two groups based on a GA level of 15.5%, the cutoff point indicating the development of early-phase diabetes. Multivariate linear regression analysis was used to assess the interaction of GA with clopidogrel antiplatelet therapy.

**Results:**

A total of 1021 participants were evaluated, and 28.3% of patients (289 of 1021) had low responsiveness to clopidogrel assessed by TEG. In patients with elevated GA levels, low responsiveness to clopidogrel assessed by TEG was observed in 33.7% (139 of 412) of patients, which was a significantly higher rate than that in the lower-GA-level group (24.6%, P = 0.002). According to multivariate linear regression analysis, a GA level > 15.5% was independently associated with low responsiveness to clopidogrel after adjustment for age, sex and other conventional confounding factors. This interaction was not mediated by a history of diabetes mellitus. A GA level ≤ 15.5% was associated with a high positive value [75.4%, 95% CI 73.0–77.6%] for predicting a normal responsiveness to clopidogrel.

**Conclusions:**

GA could be a potential biomarker to predict the effects of clopidogrel antiplatelet therapy in ACS patients and might be a clinical biomarker to guide DAPT de-escalation.

## Background

Dual antiplatelet therapy (DAPT) with aspirin and a type of P2Y12 inhibitor is the cornerstone of preventive treatment for patients who present with acute coronary syndrome (ACS) and undergo percutaneous coronary intervention (PCI), and it has been recommended by guidelines to prevent thrombotic complications and improve prognosis [1–3]. In ACS patients, although ticagrelor and prasugrel are recommended over clopidogrel [3], clopidogrel is still used widely because it is associated with a lower risk of major bleeding [4, 5] and lower financial burden [6].

However, the pharmacodynamic effects of clopidogrel are influenced by many clinical and genetic factors [7–9], and a considerable number of patients are poorly responsive to clopidogrel and have a higher risk of thrombotic events than normal responders [10, 11]. Therefore, while DAPT de-escalation in ACS patients may be considered an alternative treatment regimen to reduce bleeding risk and/or cost, some patients may be at risk for thrombotic complications [12, 13]. On the basis of which guideline is being followed, de-escalation may be performed based on clinical judgment alone, or it may be guided by platelet function testing or CYP2C19 genotyping [13]. Clinical markers are useful tools to help the doctors to make correct clinical judgments and select the most appropriate strategy of treatment.

Patients with diabetes mellitus have been found to be less sensitive to clopidogrel therapy than nondiabetic patients [14, 15], but hemoglobin A1c (HbA1c), the standard measure of long-term (2–3 months) glucose control, may not be a good predictor of low responsiveness to clopidogrel [16–18]. Glycated albumin (GA), a marker of shorter-term (2–4 weeks) glycemic control, is strongly associated with the occurrence of diabetes [19]. Consequently, GA levels might be useful to predict ACS patients’ response to antiplatelet therapy and be a good clinical biomarker to guide DAPT de-escalation.

The aim of the present study was to investigate the contributions of GA to platelet P2Y12 inhibition induced by clopidogrel as assessed by thromboelastography (TEG) and light transmittance aggregometry (LTA) in patients with ACS. Both platelet function tests can be used to calculate the quantitative platelet inhibition rate to determine low responsiveness to clopidogrel [20, 21].

## Methods

### Study design and patients

All patients with ACS who received a maintenance dose of clopidogrel (75 mg, once daily) and aspirin (100 mg, once daily) in-hospital and out-hospital were consecutively recruited in the Department of Cardiology, Beijing Anzhen Hospital, Capital Medical University, from March 2018 to July 2019. Clopidogrel was given as either a dose of 300 mg at least 12 h before platelet function testing or a dose of 75 mg for at least 5 days before platelet function testing [3, 18, 20]. The major exclusion criteria included the following: patient age < 18 years old, symptoms of severe heart failure (New York Heart Association class III and above), severely impaired liver or renal function before the procedure (serum alanine aminotransferase > 2.5 times the upper limit of normal, serum creatinine > 2.0 mg/dL), known contraindication to aspirin or clopidogrel treatment, a history of bleeding diathesis or gastrointestinal bleeding, and a history of cerebral hemorrhage or cavum subarachnoid bleeding. The present study protocol complied with the Declaration of Helsinki and was approved by the Institutional Review Board (IRB number: 2018075X) of Beijing Anzhen Hospital. Written informed consent was obtained from all participants or their legal proxies.

### Platelet function tests: thromboelastography and light transmittance aggregometry

Blood samples for platelet function tests were collected at least 12 h after the patient had taken the clopidogrel dose to ensure full antiplatelet effects. Peripheral venous whole blood was drawn by venipuncture into vacutainer tubes containing 3.2% trisodium citrate and lithium heparin (Becton–Dickinson, San Jose, CA).

A detailed description of thromboelastography has been outlined previously [21, 22]. Briefly, blood sample measurements were performed using the CFMS TEG System (LEPU Medical, Beijing, China) to detect the effects of antiplatelet therapy action via the arachidonic acid (AA) and adenosine diphosphate (ADP) pathways. The CFMS TEG System (LEPU Medical, Beijing, China) relied on the measurement of thrombin-induced clot strength to enable a quantitative analysis of platelet function. The assay incorporated the use of heparin as an anticoagulant to eliminate thrombin activity in the sample. Reptilase and factor XIIIa (activator F) were used to generate a cross-linked fibrin clot to isolate the fibrin contribution to clot strength. The contribution of P2Y12 receptor pathways to clot formation could be measured by the addition of the appropriate agonist, AA or ADP. Blood was analyzed according to the manufacturer’s instructions. One milliliter heparinized blood was transferred to a vial containing kaolin and mixed by inversion. Five hundred microliters of the activated blood were then transferred to a vial containing heparinase and mixed to neutralize heparin. The neutralized blood (360 μL) was immediately added to a heparinase-coated cup and assayed in the TEG System to measure the maximum amplitude of thrombin-induced clot strength (MA_thrombin_). Heparinized blood (340 μL) was added to a noncoated cup containing reptilase and activator F to generate a whole blood crosslinked clot in the absence of thrombin generation or platelet stimulation (MA_fibrin_). A third sample (340 μL) of heparinized blood was added to a nonheparinase-coated cup in the presence of the activator F and ADP (2 μmol) or AA (1 mmol/L) to generate a whole blood-crosslinked clot with platelet activation (MA_ADP_ or MA_AA_). ADP-induced platelet inhibition was calculated with computerized software on the basis of the formula: ADP-induced platelet inhibition rate = $(1-\frac{{\mathrm{MA}}_{\mathrm{ADP}}-{\mathrm{MA}}_{\mathrm{fibrin}}}{{\mathrm{MA}}_{\mathrm{thrombin}}-{\mathrm{MA}}_{\mathrm{fibrin}}})\times$ 100%. Low responsiveness to clopidogrel was defined as the maximum amplitude of ADP-induced platelet-fibrin clot strength (MA_ADP_) > 47 mm plus an ADP-induced platelet inhibition rate < 50% [20, 23].

The measurement of platelet aggregation by light transmittance aggregometry was also assessed as described previously [24]. The blood-citrate tubes were centrifuged at 120*g* for 5 min to recover platelet-rich plasma and further centrifuged at 850*g* for 10 min to recover platelet-poor plasma. Platelet-rich plasma and platelet-poor plasma were used as a reference for establishing the 100% and baseline optical density, respectively. ADP (Rolf Greiner Biochemica, Flacht, Germany)-induced platelet aggregation was assessed in a 20 μmol/L solution using a ChronoLog aggregometer (ChronoLog Model 700; ChronoLog, Havertown, Pennsylvania, USA). Aggregation was expressed as the maximum percent change in light transmittance from baseline. The normal range of ADP-induced platelet aggregation was 50–70% in our center, and low responsiveness to clopidogrel was defined as the ADP-induced platelet aggregation > 40%, which was in agreement with that of a previous study [24].

### Measurement of GA level

Venous blood was drawn from fasting patients by venipuncture into vacutainer tubes with no additives (Becton–Dickinson, San Jose, CA). The GA assay (Lucica GA-L glycated albumin assay kit, Asahi Kasei Pharma Corporation, Japan) was performed in the clinical laboratory in Anzhen Hospital using an automated biochemical analyzer (Beckman AU5400 Chemistry System), and the results are expressed as the ratio of glycated albumin to albumin.

### Demographic, clinical and laboratory assessments

Demographic variables included age and sex. Behavioral risk factors included body mass index (BMI, weight in kilograms divided by the square of height in meters) and cigarette smoking. Clinical and laboratory data included (1) presentations of ACS: unstable angina (UA), non-ST-segment elevation myocardial infarction (non-STEMI), and ST-segment elevation myocardial infarction (STEMI); (2) medical history of diabetes mellitus, hypertension, ischemic stroke, myocardial infarction, coronary artery bypass graft, or PCI; (3) baseline platelet count, serum lipid levels (total cholesterol [TC], low-density lipoprotein-cholesterol [LDL-C], high-density lipoprotein-cholesterol [HDL-C], triglycerides [TGs]), and creatinine levels; (4) major medications administered in the hospital: angiotensin receptor blockers (ARBs), angiotensin-converting enzyme inhibitors (ACEIs), β-blockers, calcium channel blocking agents (CCBs), statins, and proton pump inhibitors (PPIs); and (5) glucose indexes: fasting glucose, HbA1c, glycated albumin and insulin.

### Statistical analysis

Based on the hypothesis that patients with higher GA levels would exhibit an impaired response to clopidogrel, we assumed that a GA level > 15.5% was independently associated with a higher probability of low responsiveness to clopidogrel with an odds ratio (OR) = 1.8, according to a previous study assessing the relationship between ischemic stroke and GA level [25]. One previous study showed that 29.0% of patients with normal responsiveness to clopidogrel had previously or newly diagnosed diabetes mellitus [26], and we assumed that 40% of clopidogrel responders had a GA level > 15.5%. We computed the detectable ORs based on a required power of 90% and a significance level of 0.05 for comparing the rates of a GA level > 15.5% between the two kinds of clopidogrel response groups. A sample size of 492 was needed, and this procedure was performed with PASS 11 (NCSS, LLC. Kaysville, Utah, USA).

Continuous variables are presented as the mean ± standard deviation or medians with interquartile ranges and were compared using Student’s t test or Mann–Whitney U test, as appropriate. Normality was tested by the Kolmogorov–Smirnov test. Noncontinuous and categorical variables are expressed as frequencies and percentages and were compared by using the chi-square test or Fisher’s exact test, depending on the size of the analyzed group of patients. Pearson correlation analysis was carried out to assess the relationship between GA level and platelet aggregation. Spearman correlation analysis was performed to assess the relationship between GA level and medical history of diabetes mellitus. Multivariate linear regression analysis with calculation of ORs was used to test the independent contribution of each covariate to the responsiveness to clopidogrel. ORs and 95% confidence intervals (CIs) were calculated. Adjustments were made for possible confounding effects, including baseline demographic characteristics (age, sex, BMI), smoking status, comorbidities (diabetes mellitus, hypertension, presentations of ACS), and laboratory examinations (platelet count, TC, LDL-C, HDL-C, creatinine and GA). A 2-sided P value of < 0.05 was considered statistically significant. All statistical analyses were performed with SPSS Statistics 20.0 (SPSS, Inc., Chicago, IL, USA).

## Results

### Study population

A total of 1021 consecutive clopidogrel-treated ACS patients were enrolled in the study. There were no missing baseline data for variables of interest. The baseline characteristics of all patients are detailed in Table 1. As shown in Table 1, 28.3% of patients (289 of 1021) had low responsiveness to clopidogrel. According to univariate analysis, they were more often female and older, and less likely to be current or former smokers. They more often had a history of hypertension and coronary artery bypass graft; had a higher baseline platelet count and levels of TC, LDL-C, and HDL-C; and a lower baseline level of creatinine. Furthermore, they had higher levels of GA (16.6 ± 3.6% vs. 15.6 ± 3.4%, P = 0.000) but did not have higher levels of fasting plasma glucose or HbA1c (6.4 ± 2.0 mmol/L vs. 6.2 ± 2.3 mmol/L, P = 0.154; 6.3 ± 1.2% vs. 6.5 ± 1.3%, P = 0.093).

**Table 1:** Demographic and clinical characteristics of the ACS patients by responsiveness to clopidogrel assessed by thromboelastography

	Responders to clopidogrel (n = 732)	Nonresponders to clopidogrel (n = 289)	P value
Male, m (%)	601 (82.1%)	151 (52.2%)	0.000
Age, y	59.2 ± 10.3	62.9 ± 9.8	0.000
BMI, kg/m^2^	25.9 ± 3.2	25.6 ± 3.2	0.207
Medical history, n, (%)			
Diabetes mellitus	247 (33.7%)	113 (39.1%)	0.107
Hypertension	463 (63.3%)	205 (70.9%)	0.020
Previous stroke	74 (10.1%)	39 (13.5%)	0.120
Previous myocardial infarction	55 (7.5%)	22 (7.6%)	0.957
Previous CABG	3 (0.4%)	5 (1.7%)	0.045
Previous PCI	53 (8.7%)	27 (6.6%)	0.236
Presentations of ACS			0.496
Unstable angina	669 (91.4%)	258 (89.3%)	
Non-STEMI	32 (4.4%)	14 (4.8%)	
STEMI	31 (4.2%)	17 (5.9%)	
Current or previous smoking, n (%)	440 (60.1%)	117 (40.5%)	0.000
Baseline laboratory evaluation			
PLT, × 10^9^/L	210.5 ± 55.2	234.4 ± 62.1	0.000
TC, mmol/L	3.8 ± 0.9	4.1 ± 1.0	0.000
LDL-C, mmol/L	2.2 ± 0.7	2.4 ± 0.8	0.001
HDL-C, mmol/L	1.0 ± 0.2	1.1 ± 0.3	0.002
TGs, mmol/L	1.6 ± 1.0	1.5 ± 0.9	0.590
Creatinine, μmol/L	72.0 ± 14.8	68.1 ± 17.2	0.000
Major medication administered in hospital			
ARBs, n, (%)	110 (15.0%)	40 (13.8%)	0.630
ACEIs, n, (%)	161 (22.0%)	77 (26.2%)	0.113
β-blockers, n, (%)	510 (69.7%)	209 (72.3%)	0.404
CCBs, n, (%)	61 (8.3%)	21 (7.3%)	0.572
Statins, n, (%)	715 (97.7%)	282 (97.6%)	0.925
PPIs, n, (%)	369 (50.4%)	141 (48.8%)	0.641
Glucose indices			
Fasting glucose, mmol/L	6.2 ± 2.3	6.4 ± 2.0	0.154
HbA1c, %	6.5 ± 1.3	6.3 ± 1.2	0.093
Glycated albumin, %	15.6 ± 3.4	16.6 ± 3.6	0.000
HOMA-IR	2.40 (1.65–3.77)	2.54 (1.71–3.95)	0.513

*BMI* body mass index, *CABG* coronary artery bypass graft, *PCI* percutaneous coronary intervention, *non-STEMI* non-ST-segment elevation myocardial infarction, *STEMI* ST-segment elevation myocardial infarction, *PLT* platelet count, *TC* total cholesterol, *LDL-C* low-density lipoprotein-cholesterol, *HDL-C* high-density lipoprotein-cholesterol, *TGs* triglycerides, *ARBs* angiotensin receptor blockers, *ACEIs* angiotensin-converting enzyme inhibitors, *CCBs* calcium channel blocking agents, *PPIs* proton pump inhibitor, *HbA1c* hemoglobin A1c, *HOMA-IR* homeostatic model assessment for insulin resistance, calculated using the following formula: fasting serum insulin (μU/mL) * FPG (mmol/L)/156

### Weak antiplatelet effect of clopidogrel in patients with elevated GA

Many variables could affect patients’ responsiveness to the antiplatelet activity of clopidogrel, such as age, sex, BMI, smoking status, history of diabetes mellitus, hypertension, presentations of ACS, and baseline level of platelet count, TC, LDL-C, HDL-C, creatinine and GA. According to multiple linear regression analysis with the multivariate model, we found that age [OR: 1.028, 95% CI (1.011 to 1.046), P = 0.002], female sex [OR: 2.964, 95% CI (1.869 to 4.700), P = 0.000], platelet count [OR: 1.006, 95% CI (1.003 to 1.009), P = 0.000] and GA > 15.5% [OR: 1.489, 95% CI (1.012 to 2.189), P = 0.043] were independently associated with low responsiveness to clopidogrel. These analyses showed that although the GA level correlated with a history of diabetes mellitus (r = 0.622, P = 0.000), the interaction of GA with clopidogrel antiplatelet therapy was not mediated by a history of diabetes mellitus.

### High ADP-induced platelet aggregation in patients with elevated GA

Light transmittance aggregometry was also implemented in the current study to assess the responsiveness to clopidogrel. As shown in Additional file 1: Table S1, 30.4% of patients (310 of 1021) had low responsiveness to clopidogrel, which was correlated with ADP-induced platelet aggregation > 40%. According to univariate analysis, patients were more often female and older and less likely to have unstable angina. They had a higher baseline level of TC (3.9 ± 1.0 mmol/L vs. 3.8 ± 1.0 mmol/L, P = 0.047) and GA (16.1 ± 3.7% vs. 15.5 ± 3.2%, P = 0.010). Multiple linear regression analysis revealed that female sex [OR: 1.496, 95% CI (1.075 to 2.083), P = 0.017] and GA > 15.5% [OR: 1.415, 95% CI (1.061 to 1.888), P = 0.018] were independently associated with low responsiveness to clopidogrel.

### GA levels and effect on antiplatelet therapy with clopidogrel

All patients included in this study were categorized into two groups based on a GA level of 15.5%. The cut-off point was chosen because it may predict the presence of early-phase diabetes [27]. As shown in Table 2, patients with elevated GA levels were more often female and older and were more likely to have diabetes mellitus. These patients had higher baseline fasting plasma glucose and HbA1c levels but lower platelet counts.

**Table 2:** Baseline characteristics and outcomes of patients by GA categories

	GA ≤ 15.5% (n = 609)	GA > 15.5% (n = 412)	P value
Male, m (%)	465 (76.4%)	287 (69.7%)	0.017
Age, y	58.4 ± 10.0	63.0 ± 10.1	0.000
BMI, kg/m^2^	26.0 ± 3.3	25.6 ± 3.1	0.126
Medical history, n, (%)			
Diabetes mellitus	73 (12.0%)	287 (69.7%)	0.000
Hypertension	386 (63.4%)	282 (68.4%)	0.095
Previous stroke	62 (10.2%)	51 (12.4%)	0.272
Previous myocardial infarction	40 (6.6%)	37 (9.0%)	0.152
Previous CABG	2 (0.3%)	6 (1.5%)	0.067
Previous PCI	50 (8.2%)	26 (6.3%)	0.257
Presentations of ACS			0.319
Unstable angina	555 (91.1%)	372 (90.3%)	
Non-STEMI	23 (3.8%)	23 (5.6%)	
STEMI	31 (5.1%)	17 (4.1%)	
Current or previous smoking, n (%)	339 (55.7%)	218 (52.9%)	0.386
Baseline laboratory evaluation			
PLT, × 10^9^/L	220.8 ± 56.6	212.0 ± 60.2	0.019
TC, mmol/L	3.9 ± 1.0	3.9 ± 0.9	0.342
LDL-C, mmol/L	2.3 ± 0.8	2.2 ± 0.7	0.356
HDL-C, mmol/L	1.1 ± 0.2	1.1 ± 0.3	0.875
TGs, mmol/L	1.6 ± 0.9	1.5 ± 1.0	0.451
Creatinine, μmol/L	71.0 ± 14.4	70.7 ± 17.2	0.734
Glucose indices			
Fasting glucose, mmol/L	5.4 ± 1.6	7.5 ± 2.4	0.000
HbA1c, %	5.9 ± 0.6	7.4 ± 1.4	0.000
HOMA-IR	2.1 (1.6–3.1)	3.3 (1.9–4.9)	0.000
Antiplatelet responsiveness to clopidogrel			
Responders to clopidogrel	459 (75.4%)	273 (66.3%)	0.002
ADPi	45.4 ± 25.4	39.7 ± 23.8	0.000
MA_ADP_	35.2 ± 14.5	39.0 ± 13.9	0.000

*BMI* body mass index, *CABG* coronary artery bypass graft, *PCI* percutaneous coronary intervention, *non-STEMI* non-ST-segment elevation myocardial infarction, *STEMI* ST-segment elevation myocardial infarction, *PLT* platelet count, *TC* total cholesterol, *LDL-C* low-density lipoprotein-cholesterol, *HDL-C* high-density lipoprotein-cholesterol, *TGs* triglycerides, *HbA1c* hemoglobin A1c, *HOMA-IR* homeostatic model assessment for insulin resistance, *ADPi* ADP-induced platelet inhibition rate, *MA*_*ADP*_ the maximum amplitude of ADP-induced platelet-fibrin clot strength

**Fig. 1:**
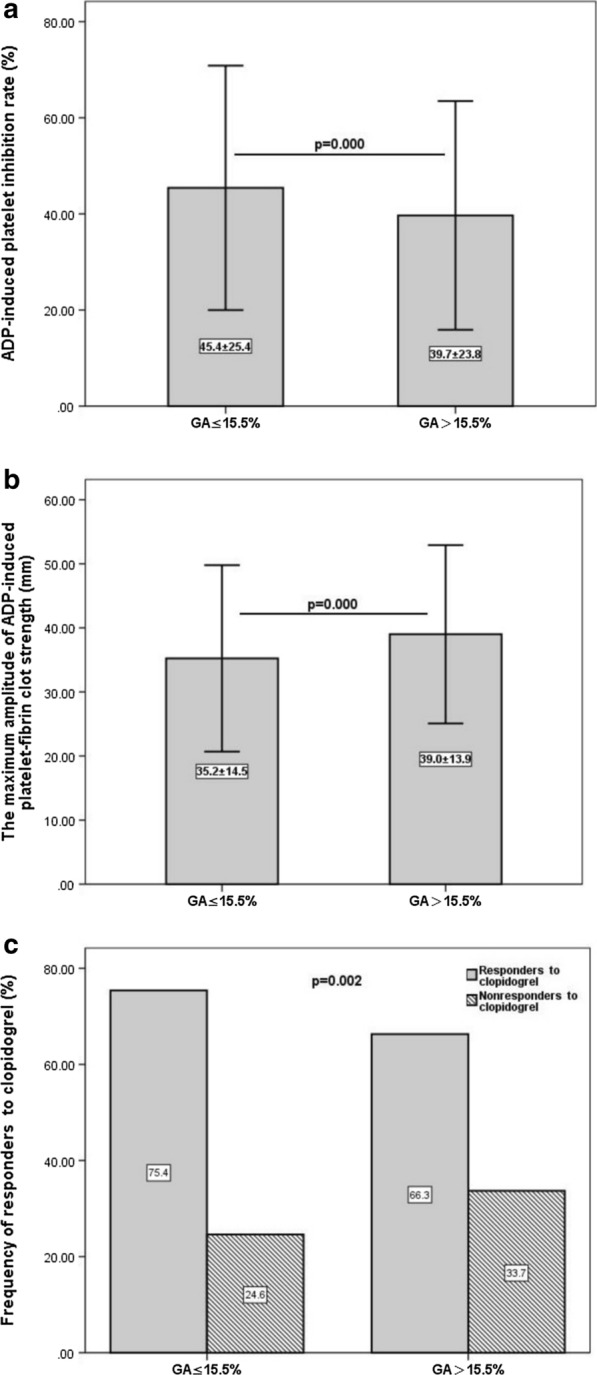
**a** ADP-induced platelet inhibition rate (ADPi) and **b** the maximum amplitude of ADP-induced platelet-fibrin clot strength (MA_ADP_) in patients with low (≤ 15.5%) and high (> 15.5%) glycated albumin (GA). **c** Analysis of the frequency of responders to clopidogrel with low/high GA level. Analysis showed patients with a GA level ≤ 15.5% had a normal response to clopidogrel more often than patients with a GA level > 15.5% (P = 0.002)

There was also a significant interaction between GA level and the antiplatelet effect of clopidogrel measured by TEG. Pearson correlation analysis revealed that the GA level was significantly negatively correlated with the ADP-induced platelet inhibition rate (r = − 0.161, P = 0.000) and positively correlated with the value of MA_ADP_ (r = 0.191, P = 0.000). Additionally, in patients with a GA level ≤ 15.5%, the ADP-induced platelet inhibition rate was higher, and the value of MA_ADP_ was obviously lower (Fig. 1a, b); that is, these patients had a normal response to clopidogrel more often than patients with a GA level > 15.5% (75.4% vs. 66.3%, P = 0.002; Fig. 1c). In this study group, a GA level ≤ 15.5% was associated with a high positive value [75.4%, 95% CI 73.0% to 77.6%] for the prediction of normal responsiveness to clopidogrel and might be a clinical biomarker to guide DAPT de-escalation.

## Discussion

Increased platelet aggregation and activation have been observed in diabetic patients compared with those in nondiabetic patients, and a high proportion of nonresponders to clopidogrel were also observed in the diabetic population [17, 28]. It has also been described that patients with poorly controlled diabetes have greater platelet reactivity and weaker response to clopidogrel [18, 29–31]. In these studies, HbA1c was the most common marker of glycemic control. However, even though an HbA1c level ≥ 6.5% has been recommended as one of the criteria for diagnosing diabetes mellitus [32], the limitations of HbA1c have been widely acknowledged. The use of HbA1c is not recommended in clinical situations that might interfere with the metabolism of hemoglobin, such as in the contexts of hemolytic, secondary or iron-deficiency anemia; hemoglobinopathies; pregnancy; and uremia [32], and HbA1c exhibits a delayed response to changes in treatment since it reflects the effect of long-term control of glycemia. GA is a new measure of glycemia based on the amount of glucose in the serum or plasma attached to albumin rather than to erythrocyte hemoglobin. GA does not require fasting for its measurement and reflects short-term glycemia due to the short half-life of albumin, which is approximately 2–4 weeks [33]. Compared to HbA1c, GA can be used for patients with anemia or hemoglobinopathies and could more accurately reflect the actual status of glycemic control [34].

### Association of antiplatelet responsiveness to clopidogrel with GA levels

The main finding of this study is the documented relationship between the GA level on admission and antiplatelet responsiveness in clopidogrel-treated ACS patients according to TEG and LTA. A GA level > 15.5% was an independent risk factor for increased platelet reactivity in the TEG and LTA test performed to assess the response to clopidogrel, and a GA level ≤ 15.5% was associated with a positive predictive value > 70% for the prediction of normal responsiveness to clopidogrel, which might be a clinical biomarker to guide DAPT de-escalation. Previous studies have demonstrated the association between GA and the effect of DAPT on ischemic stroke [25], but to the best of our knowledge, no studies have examined the interaction in ACS patients, in whom the achievement of optimal P2Y12 inhibition is of paramount importance.

Despite the greater efficacy of prasugrel and ticagrelor compared with clopidogrel in ischemic benefit after ACS and being recommended by guidelines for these patients [3], clopidogrel is still broadly used. The lower risk for ischemic events beyond the acute phase of ACS, combined with the bleeding risk associated with prolonged potent P2Y2 inhibition, has raised concern about the clinical benefit associated with their chronic use. Therefore, P2Y12 inhibitor de-escalation frequently occurs among patients with ACS in real-world practice, which can be done unguided based on clinical judgment or guided by platelet function testing or CYP2C19 genotyping [13]. However, if clinicians apply platelet function testing to guide de-escalation, they must switch patients from a potent P2Y12 inhibitor to clopidogrel and then wait for clopidogrel to achieve its steady-state effects prior to performing platelet function testing, and if low responsiveness to clopidogrel is confirmed, the therapy with a potent P2Y12 inhibitor needs to be re-instituted [35]. From a more practical clinical viewpoint, measuring blood biomarkers such as GA is less time consuming and less expensive than sequencing variants of cytochrome P450 genes. Moreover, because GA level reflects a shorter-term mean glycemia level (2–4 weeks), it remains stable and is a real marker of the glycemic control several weeks before ACS onset. Therefore, this study suggested an easy-to-implement and economical method to predict a patient’s response to clopidogrel therapy.

### Potential underlying mechanisms

Common cardiovascular risk factors, such as age, sex, BMI, smoking status, diabetes mellitus, hypertension, hypercholesterolemia, and chronic kidney disease, are known to interfere with clopidogrel antiplatelet effects [9, 36]. According to multiple linear regression analysis, we adjusted these factors and found that the independent effect of GA on antiplatelet therapy with clopidogrel remained, and this association was independent of history of diabetes mellitus. This result has various explanations. First, GA is characterized by more rapid changes than HbA1c and reflects the change in patients’ acute phase glycemia status prior to ACS onset, regardless of the history of diabetes. Second, GA with loss of fatty-acid-binding capacity could also promote platelet activation and aggregation, which contribute to platelet hyperactivity and increased thrombosis [37–39]. Finally, we used a GA value of 15.5% as the cut-off point, which was the recommended value to predict the presence of early-phase diabetes in an epidemiologic study of an East Asian population [27]. Therefore, the cut-off point we used might identify patients with diabetes at an earlier stage than currently possible. These findings were supported by the findings of a weak association between the GA level and history of diabetes.

## Limitations

Several limitations in the present study should be mentioned. This was a single-center study with a relatively small sample size, which may introduce bias into the primary findings. Second, TEG- and LTA-defined low responsiveness to clopidogrel was the primary efficacy outcome rather than clinical follow-up of cardiac death, nonfatal myocardial infarction and stroke. Third, the measurement of the GA level and platelet reactivity was performed only at baseline, and it would be more useful if serial testing were performed, as some studies have indicated that platelet responsiveness can change over time [40, 41]. Future large-scale prospectively designed trials with appropriate clinical follow-up intervals are warranted to verify the effect of GA on antiplatelet therapy with clopidogrel in ACS patients.

## Conclusion

In the current study, a high GA level was associated with lower responsiveness to clopidogrel in ACS patients, and this relationship was not mediated by a history of diabetes mellitus. Thus, our findings indicated that the GA level is a useful and practical indicator of ACS patients’ responsiveness to clopidogrel and a GA level ≤ 15.5% might be a clinical biomarker to guide DAPT de-escalation.

## Supplementary information

**Additional file 1: Table S1.** Demographic and clinical characteristics of the ACS patients by responsiveness to clopidogrel assessed by light transmittance aggregometry.

## Data Availability

The datasets used and/or analyzed during the current study are available from the corresponding author on reasonable request.

## Abbreviations

GA: Glycated albumin
ACS: Acute coronary syndrome
TEG: Thromboelastography
LTA: Light transmittance aggregometry
ADP: Adenosine diphosphate
DAPT: Dual antiplatelet therapy
PCI: Percutaneous coronary intervention
HbA1c: Hemoglobin A1c
MA_ADP_: The maximum amplitude of ADP-induced platelet-fibrin clot strength
BMI: Body mass index
UA: Unstable angina
Non-STEMI: Non-ST-segment elevation myocardial infarction
STEMI: ST-segment elevation myocardial infarction
TC: Total cholesterol
LDL-C: Low-density lipoprotein-cholesterol
HDL-C: High-density lipoprotein-cholesterol
TGs: Triglycerides
ARBs: Angiotensin receptor blockers
ACEIs: Angiotensin-converting enzyme inhibitors
CCBs: Calcium channel blocking agents
PPIs: Proton pump inhibitors
OR: Odds ratio
CIs: Confidence intervals
CABG: Coronary artery bypass graft
PLT: Platelet count
HOMA-IR: Homeostatic model assessment for insulin resistance
