# Supplementary material for: High glycated albumin is an independent predictor of low response to clopidogrel in ACS patients: a cross-sectional study
Source: Cardiovasc Diabetol. 2020 Oct 9;19:171. doi: 10.1186/s12933-020-01146-w (PMC7545941; doi:10.1186/s12933-020-01146-w)
Supplement: Supplementary file 1 — Additional file 1: Table S1. Demographic and clinical characteristics of the ACS patients by responsiveness to clopidogrel assessed by light transmittance aggregometry. [file 12933_2020_1146_MOESM1_ESM.docx]

**Table S1 Demographic and clinical characteristics of the ACS patients by responsiveness to clopidogrel assessed by light transmittance aggregometry**

|  | Responders to clopidogrel(n = 711) | Nonresponders to clopidogrel(n = 310) | P Value |
| --- | --- | --- | --- |
| Male, m (%) | 570(80.2%) | 182(58.7%) | 0.000 |
| Age, y | 59.1±10.5 | 60.8±10.2 | 0.015 |
| BMI, kg/m^2^ | 25.7±3.2 | 25.9±3.2 | 0.270 |
| Medical history, n, (%) |  |  |  |
| Diabetes mellitus | 264(37.1%) | 96(31.0%) | 0.058 |
| Hypertension | 473(66.5%) | 195(62.9%) | 0.263 |
| Previous stroke | 86(12.1%) | 27(8.7%) | 0.113 |
| Previous myocardial infarction | 51(7.2%) | 26(8.4%) | 0.499 |
| Previous coronary artery bypass graft | 6(0.8%) | 2(0.6%) | 1.000 |
| Previous percutaneous coronary intervention | 59(8.3%) | 21(6.8%) | 0.405 |
| Presentations of ACS |  |  | 0.030 |
| Unstable angina | 654(92.0%) | 273(88.1%) |  |
| Non–STEMI | 24(3.4%) | 22(7.1%) |  |
| STEMI | 33(4.6%) | 15(4.8%) |  |
| Current or previous smoking, n (%) | 377(53.0%) | 180(58.1%) | 0.137 |
| Baseline laboratory evaluation |  |  |  |
| PLT, ×10^9^/L | 212.1±57.9 | 219.5±58.3 | 0.063 |
| Total cholesterol, mmol/L | 3.8±1.0 | 3.9±1.0 | 0.047 |
| LDL-C, mmol/L | 2.2±0.8 | 2.3±0.7 | 0.087 |
| HDL-C, mmol/L | 1.0±0.2 | 1.1±0.3 | 0.172 |
| Triglycerides, mmol/L | 1.5±0.9 | 1.5±0.9 | 0.220 |
| Creatinine, μmol/L | 71.6±15.9 | 70.6±15.5 | 0.315 |
| Major medication administered in hospital |  |  |  |
| ARBs, n, (%) | 109(15.3%) | 41(13.2%) | 0.278 |
| ACEIs, n, (%) | 159(22.4%) | 79(25.5%) | 0.382 |
| β-blockers, n, (%) | 505(71.0%) | 214(69.0%) | 0.521 |
| CCBs, n, (%) | 52(7.3%) | 30(9.7%) | 0.201 |
| Statins, n, (%) | 695(97.7%) | 302(97.4%) | 0.749 |
| Proton pump inhibitor, n, (%) | 360(50.6%) | 150(48.4%) | 0.509 |
| Glucose indices |  |  |  |
| Fasting plasma glucose, mmol/L | 6.1±1.9 | 6.3±2.3 | 0.068 |
| HbA1C, % | 6.4±1.2 | 6.6±1.3 | 0.066 |
| Glycated albumin, % | 15.5±3.2 | 16.1±3.7 | 0.010 |
| HOMA-IR | 2.30(1.72-3.61) | 2.47(1.67-3.93) | 0.485 |

BMI:body mass index; CABG: coronary artery bypass graft; PCI: percutaneous coronary intervention; non-STEMI: non–ST-segment elevation myocardial infarction; STEMI: ST-segment elevation myocardial infarction; PLT: platelet count; TC: total cholesterol; LDL-C: low-density lipoprotein-cholesterol; HDL-C: high-density lipoprotein-cholesterol; TGs: triglycerides; ARBs: angiotensin receptor blockers; ACEIs: angiotensin-converting enzyme inhibitors; CCBs: calcium channel blocking agents; PPIs: proton pump inhibitor; HbA1c: hemoglobin A1c; HOMA-IR: homeostatic model assessment for insulin resistance.
